# Case Report: Is It Premature Ovarian Insufficiency or Swyer Syndrome After Bone Marrow Transplantation?

**DOI:** 10.3389/fped.2021.808277

**Published:** 2022-01-13

**Authors:** Hui Li, Jin Li, Xiaohong Li, Hong Yi, Qixiu Ren, Xiaoyan Chen

**Affiliations:** ^1^Department of Reproductive Health, Shenzhen Baoan Women's and Children's Hospital, Shenzhen University, Shenzhen, China; ^2^Department of Obstetrics and Gynaecology, Shenzhen Baoan Women's and Children's Hospital, Shenzhen University, Shenzhen, China; ^3^Department of Obstetrics and Gynaecology, The Chinese University of Hong Kong, Hong Kong, Hong Kong SAR, China

**Keywords:** premature ovarian insufficiency (POI), bone marrow transplantation, thalassemia major, primary amenorrhea, 46, XY karyotype

## Abstract

**Introduction:** Iatrogenic factor is one of the recognized causes for premature ovarian insufficiency. The aim of this case report was to present a rare case with premature ovarian insufficiency and 46, XY karyotype after bone marrow transplant (BMT) for thalassaemia major at childhood. We also reviewed some relevant literature in this report.

**Case Presentation:** A 17-year-old girl was presented with primary amenorrhea and premature ovarian insufficiency after receiving chemotherapy and BMT from her brother due to thalassaemia major at childhood. She had poor secondary sex characteristics, assessed as stage I for the development of breasts and external genitalia based on the Tanner scale. Transabdominal ultrasound showed small uterus with visible endometrial lining and small ovaries. Laboratory data showed hypergonadotropic hypogonadism profile with low level of estrogen and high level of follicular-stimulating hormone (FSH). Patient's peripheral lymphocytes karyotype was 46, XY.

**Conclusions:** This case was diagnosed as a chemotherapy induced premature ovarian insufficiency. Patient's peripheral lymphocytes karyotype (46, XY) after she received BMT from a male donor was a misleading finding, and the case could be easily misdiagnosed as Swyer syndrome. A correct diagnosis in such cases should depend not only on the recent clinical findings, but also on the detailed medical history. To prevent premature ovarian insufficiency in similar cases, fertility preservation should be offered to girls before they receive chemotherapy, total body irradiation and BMT.

## Introduction

Premature ovarian insufficiency affects around 1% of women under the age of 40. It usually presents as oligo- or amenorrhea for at least 4 months and FSH level over 40 IU/ml, checked twice at least 4 weeks apart (1, 2). When serious ovarian function depletion occurs in childhood, most patients will experience primary amenorrhea.

Iatrogenic factors are one of the recognized causes for premature ovarian insufficiency, including radiation treatment andchemotherapy. The use of chemotherapy, radiotherapy and bone marrow transplantation (BMT) to treat malignant and nonmalignant diseases in girls and young women has become more common (3). Therefore, the awareness of fertility preservation for this group of subjects should be emphasized since the late side effects of BMT, though usually not life threatening, may significantly impair quality of life in adults (3), in whom gonadal failure is a common long-term endocrine consequence of BMT.

In this case report, we present a patient with primary amenorrhea and premature ovarian insufficiency after chemotherapy and BMT due to thalassaemia major, whose peripheral lymphocytes karyotype result (46, XY) could lead to a misdiagnosis of disorders of sexual differentiation.

## Case Presentation

This report was approved by the local hospital Ethics Committee. Patient was a 17-year-old girl who attended our outpatient clinic for evaluation of primary amenorrhea. Her past medical history revealed short period of chemotherapy followed by BMT therapy due to thalassaemia major at the age of eight. She reported to be free of the disease currently and did not take any medication. As an adolescent, the patient came with her mother, reported a lack of menstrual flow and was presented with poor secondary sex characteristics.

**Figure 1:**
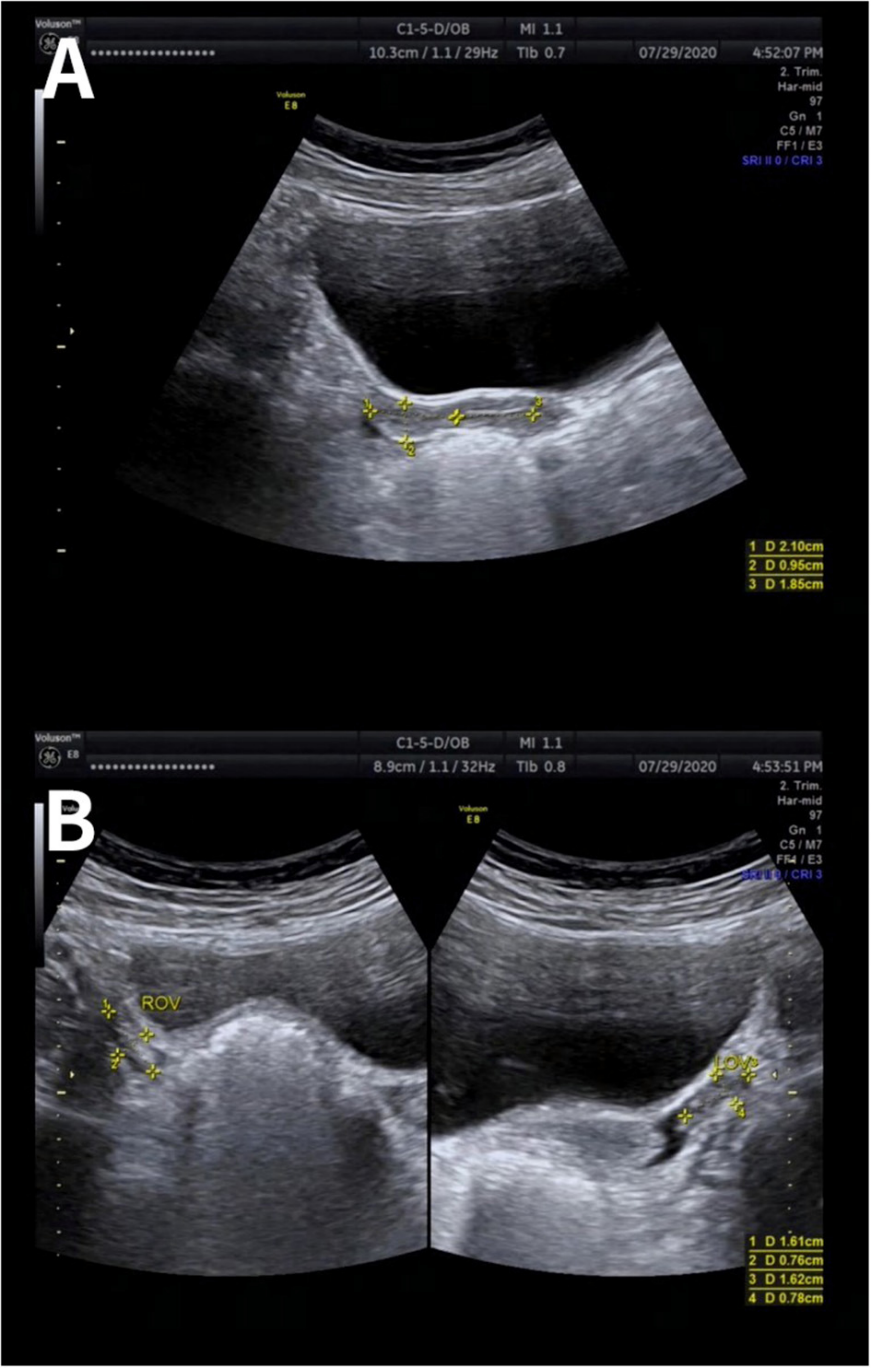
Ultrasonography image of uterus and ovaries. Transabdominal ultrasound showed small uterus (2.1 × 1.5 × 1.0 cm) with visible endometrial lining **(A)**. Both ovaries were measured as 1.6 × 0.8 cm **(B)**.

**Figure 2:**
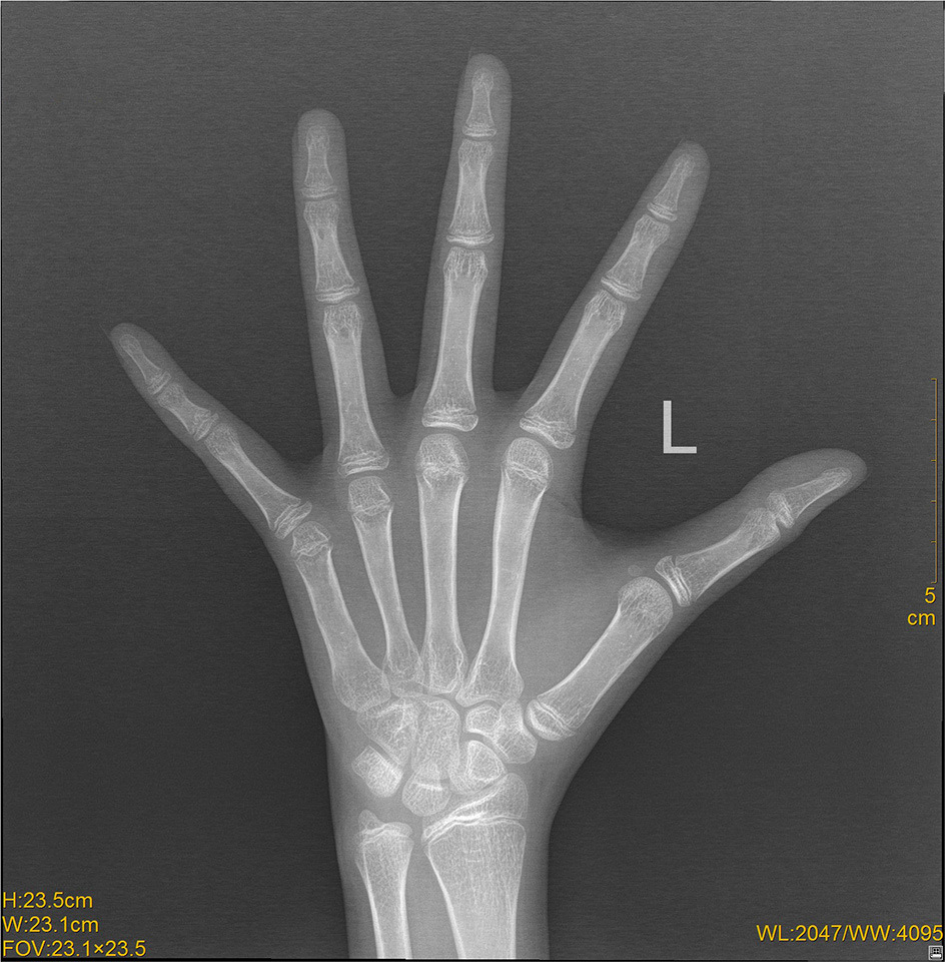
X-ray image of the left wrist. X-ray showed that the bone age was 13 years old.

During the visits, she was assessed as stage I for the development of breasts and external genitalia according to the Tanner scale. The presence of a vaginal opening was found by gynecological examination. Transabdominal ultrasound showed small uterus (2.1 × 1.5 × 1.0 cm) with visible endometrial lining; both ovaries were measured as 1.6 × 0.8 cm (Figure 1). Laboratory data showed low concentration of estradiol (<55 pmol/l) and significantly elevated levels of serum follicle-stimulating hormone (FSH) and luteinizing hormone (LH) (125.93 and 31.59 mIU/ml, respectively). Anti-Mullerian hormone (AMH) concentration was extremely low (0.04 ng/ml). The remainder of the results, including levels of basal thyroid hormones, TSH, cortisol, 17-hydroxyprogesterone and prolactin, were within the reference range. X-ray of the left wrist showed that the bone age was 13 years old (Figure 2). Patient's peripheral lymphocytes karyotype was 46, XY. A more detailed history was then taken and it was found that the patient had BMT from her brother. Based on the above characteristics, the patient was diagnosed as primary amenorrhea and premature ovarian insufficiency. A more detailed history was then taken and the entire medical record was checked carefully. When the patient was 8 years old, she had HLA-matched bone marrow hematopoietic stem cell from her brother. Before BMT, she had bone marrow aspiration and bone marrow cell karyotype was shown as 46, XX. However, when the patient came to our clinic, she refused to reassess the karyotype using other different cells, such as skin cells. Based on the above characteristics, the patient was diagnosed as primary amenorrhea and premature ovarian insufficiency.

Currently, the patient receives low dose of estrogen therapy (0.25 mg estradiol valerate orally daily) to allow the growth and development of breast and reproductive organs as well as the skeleton. The patient will be followed-up every 3 months for regular checkups in our outpatient clinic. When there is improvement of the uterus size and endometrial thickness, hormone replacement treatment using estradiol and norgestrel may be given to induce menstruation.

## Discussion and Conclusion

In females, menarche is the most significant corporal change during adolescence. Primary amenorrhea is defined as the absence of menstruation by the age of 15 with developed secondary sexual characteristics or 3 years after thelarche (4). There are several classifications of causes of primary amenorrhea, including anatomic defects, primary hypogonadism, hypothalamic causes, pituitary causes, chromosomal abnormalities and other endocrine gland disorders (5).

The prevalence of 46, XY karyotype in females with primary amenorrhea, either with pure gonadal dysgenesis or complete androgen insensitivity syndrome, is around 3–6% (6). Pure gonadal dysgenesis, also known as Swyer syndrome, may be recognized when there are not normally differentiated testes despite male karyotype. It has been reported that the mutation in several genes is responsible for Swyer syndrome, among which SRY appears to be the most significant one and responsible for 10–20% of cases (7). Patients with Swyer syndrome are typically tall-statured and with female genitalia. Another feature is hypergonadotropic hypogonadism with low levels of estrogen and AMH and high level of FSH, which results in poor breast development, amenorrhea, increased risk of cardiovascular disease (7). With regards to complete androgen insensitivity syndrome, tissues are not reactive to testosterone due to the mutation in gene encoding androgen receptor, although testes are developed and their hormone secretion function is preserved. Affected individuals are with the external female phenotype and genitalia due to the normal conversion of excessive testosterone to estrogen. However, Mullerian derivatives are not preserved and testes may be situated either in the abdominal cavity or throughout the inguinal canal or in the labia majora. Hormonal profiles usually show elevated testosterone and detectable estrogen concentrations (8).

The clinical features in our patient presented in this report, including female external genitalia, presence of uterus, hypergonadotropic hypogonadism, low AMH level and 46, XY karyotype, can be easily misdiagnosed as Swyer syndrome. However, when digging the past history of the patient, it was found that the appearance of 46, XY karyotype in the peripheral blood in this patient is due to the BMT from a male donor. In addition, her karyotype was 46, XX in the bone marrow cells before BMT. This clue can help to rule out the diagnosis of Swyer syndrome. There are two similar cases reported previously. One study reported a childhood cancer survivor with premature ovarian insufficiency and 46, XY karyotype in lymphocytes after chemotherapy and BMT from an unrelated male donor, and the karyotype appeared to be 46, XX in the swab sample from the cheek, which contained fibroblasts (9). The other report showed a patient with primary amenorrhea and 46, XY karyotype after receiving BMT from her brother and chemotherapy due to acute myeloid leukemia (10). The authors from both reports also found a high likelihood of misdiagnosis of Swyer syndrome, which was similar to our report. Therefore, the comprehensive collection of detailed present and past medical history is important for a correct diagnosis. Moreover, although the patient in this report refused to check karyotype in other different cells other than peripheral lymphocytes, it is still strongly recommended using other cells (such as skin cells) to reassess karyotype after BMI in similar cases to confirm the diagnosis.

In this case presented, several possible causes may be contributing to the observed iatrogenic premature ovarian insufficiency. After repeated red blood cell transfusions, patients with thalassemia major may have ovarian impairment due to iron overload, when the transferrin-dependent system is inhibited through ferritin saturation pathway and excessive iron accumulation occurs through the non-transferrin bound iron (NTBI) pathway (11). Although the most common endocrinopathy in patients with thalassemia is the hypogonadism resulting from iron deposition in the hypothalamic and/or pituitary cells (12), it is also worth noting that iron overload may affect ovarian function directly as well. An earlier study has shown an inverse correlation between AMH level and NTBI (13), suggesting suspected ovarian tissue iron overload in women with thalassemia major. Another study also demonstrated that AMH level and antral follicle count are significantly decreased in women with transfusion-dependent thalassemia major compared with age-matched controls (12). These findings support a deleterious effect of iron overload on ovarian tissue, which may result in an increase in reactive oxygen species and the subsequent acceleration in follicular aging (14). An earlier study has found high redox activity in the ovarian follicular fluid from a woman with thalassemia major, which suggested that redox-active iron ions may mediate free radical production and induce ovarian tissue injury (15). Therefore, there is a need to better define the appropriate chelation regimens and antioxidant supplementations regarding reproductive function in women with thalassemia major receiving blood transfusion treatment. However, whether ovarian function is impaired by a direct effect of iron overload is not clearly understood yet.

Another important factor responsible for the ovarian impairment in this case may be BMT, including chemotherapy and/or total body irradiation (TBI). The extent of follicular impairment can be affected by the type of TBI protocol as well as the age of the patient receiving BMT. In terms of chemotherapy, busulfan appears to be the most gonadotoxic regimen, with reported prevalence of premature ovarian failure as high as 100% (16, 17). Low-dose cyclophosphamide (200 mg/kg) is considered to be much less gonadotoxic compared with other regimens, with good recovery rates of clinical ovarian function, particularly in women younger than 25 years old (18, 19). In contrast, TBI appears to be a more toxic treatment prior to BMT and most patients undergoing TBI experience gonadal failure. Compared with TBI administration after puberty, TBI administered prior to puberty is reported to be less gonadotoxic and around 40–60% of patients experienced spontaneous puberty (20, 21). This might be due to the higher number of non-growing follicles found in younger girls and some other particular anatomical or paracrine factors, which might lead to a higher resistance to fibrosis implicated in the mechanisms of ovarian damage (22).

Therefore, fertility preservation is crucial for this cohort of patients, particularly prior to repeated blood transfusion treatment and BMT. In post-pubertal women, fertility preservation methods consist of oocyte freezing, embryo freezing, ovarian tissue cryopreservation and GnRH agonist application (23), while in pre-pubertal girls, ovarian tissue cryopreservation is the main option, although ovarian shielding from radiotherapy may also be available considerations (24). Ovarian cortical tissue, which contains a large reserve of oocytes in the primordial follicles, can be frozen, stored and then re-implanted after BMT treatment and pregnancy can be then possibly achieved, either naturally or by assisted reproductive technique (25). Whereas, restoration of fertility is the primary indication for re-implantation of ovarian tissue, the ovary is also an endocrine organ and restoration of hormonal function may also be an indication (26). However, ovarian tissue cryopreservation is an invasive and still experimental procedure for young girls, which requires laparoscopic surgery. Hence, more long-term follow-up data will be needed to verify the effect of ovarian tissue cryopreservation in this cohort of patients.

To conclude, this is a case report of a patient presenting with primary amenorrhea, premature ovarian insufficiency and 46, XY karyotype in peripheral blood after receiving chemotherapy and BMT from her brother in childhood due to thalassemia major, which can be easily misdiagnosed as Swyer syndrome. It is important that gynecologists be aware that not only the clinical manifestations, but also the detailed medical history, are crucial for a correct diagnosis and the following treatment. In light of the relatively high prevalence of thalassemia, particularly in the southern China, fertility preservation should be considered for young girls who are going to have chemotherapy, total body irradiation and BMT.

## Data Availability Statement

The raw data supporting the conclusions of this article will be made available by the authors, without undue reservation.

## Ethics Statement

The studies involving human participants were reviewed and approved by Ethics Committee of the Institutional Review Board (IRB) of Shenzhen Baoan Woman's and Children's Hospital. Written informed consent to participate in this study was provided by the participants' legal guardian/next of kin.

## Author Contributions

HL, JL, and XC: substantial contribution to the conception and design of the work and manuscript drafting. HL, XL, HY, and QR: performed investigations on the patient. HL and XC: participation in acquisition of the literature. All authors have read and approved the final article.

## Funding

This study was supported by Shenzhen Key Medical Discipline Construction Fund (SZXK028) and Basic and Applied Basic Research Foundation of Guangdong Province of China (2020A1515110082).

## Conflict of Interest

The authors declare that the research was conducted in the absence of any commercial or financial relationships that could be construed as a potential conflict of interest.

## Publisher's Note

All claims expressed in this article are solely those of the authors and do not necessarily represent those of their affiliated organizations, or those of the publisher, the editors and the reviewers. Any product that may be evaluated in this article, or claim that may be made by its manufacturer, is not guaranteed or endorsed by the publisher.
